# Diabetes Prevention in the New York City Sikh Asian Indian Community: A Pilot Study

**DOI:** 10.3390/ijerph110505462

**Published:** 2014-05-19

**Authors:** Nadia S. Islam, Jennifer M. Zanowiak, Laura C. Wyatt, Rucha Kavathe, Hardayal Singh, Simona C. Kwon, Chau Trinh-Shevrin

**Affiliations:** 1 Health Promotion and Prevention Research Center, Department of Population Health, School of Medicine, New York University, New York, NY 10016, USA; E-Mails: jennifer.zanowiak@nyumc.org (J.M.Z.); simona.kwon@nyumc.org (S.C.K.); chau.trinh@nyumc.org (C.T.-S.); 2 Center for the Study of Asian American Health, Department of Population Health, School of Medicine, New York University, New York, NY 10016, USA; E-Mail: laura.wyatt@nyumc.org; 3 UNITED SIKHS, Community Education & Empowerment Directorate, New York, NY 10116, USA; E-Mails: rucha.kavathe@unitedsikhs.org (R.K.); hardayal.singh@unitedsikhs.org (H.S.)

**Keywords:** Asian American, Asian Indian, community health worker, diabetes, Diabetes Prevention Program, Sikh, South Asian American

## Abstract

India has one of the highest burdens of diabetes worldwide, and rates of diabetes are also high among Asian Indian immigrants that have migrated into the United States (U.S.). Sikhs represent a significant portion of Asian Indians in the U.S. Diabetes prevention programs have shown the benefits of using lifestyle intervention to reduce diabetes risk, yet there have been no culturally-tailored programs for diabetes prevention in the Sikh community. Using a quasi-experimental two-arm design, 126 Sikh Asian Indians living in New York City were enrolled in a six-workshop intervention led by community health workers. A total of 108 participants completed baseline and 6-month follow-up surveys between March 2012 and October 2013. Main outcome measures included clinical variables (weight, body mass index (BMI), waist circumference, blood pressure, glucose, and cholesterol) and health behaviors (changes in physical activity, food behaviors, and diabetes knowledge). Changes were significant for the treatment group in weight, BMI, waist circumference, blood pressure, glucose, physical activity, food behaviors, and diabetes knowledge, and between group differences were significant for glucose, diabetes knowledge, portion control, and physical activity social interaction. Retention rates were high. Findings demonstrate that a diabetes prevention program in the Sikh community is acceptable, feasible, and efficacious.

## 1. Introduction

Diabetes, a group of diseases marked by high levels of blood glucose, can lead to serious complications and morbidity. South Asians (representing individuals from India, Pakistan, Bangladesh, and other parts of the South Asian subcontinent) have elevated risk of developing diabetes compared to other groups, and India has the second highest number of people with diabetes worldwide [1]. Recent studies have found that rates of diabetes among South Asians in the U.S. are higher than other Asian groups and the general population [2,3,4], with diabetes prevalence ranging from 14% to 35% [5,6,7,8]. Similarly, a study from New York City (NY, USA) found that foreign-born South Asians were 4.88 times more likely to have diabetes when compared to Whites [8].

The Diabetes Prevention Program (DPP) is an evidence-based program developed in the U.S. which has demonstrated effectiveness in preventing type 2 diabetes [9], and has been found to be more effective than metformin [10]. Diabetes is preventable through dietary changes and weight loss, increased physical activity, and other lifestyle changes [10,11], and in addition to lowering clinical measurements such as weight and glucose [11,12,13,14,15,16,17,18,19,20,21,22,23], community-based interventions modeled after the DPP have also been shown to promote health benefits such as improved nutrition and increased exercise [12,13,15,22,23,24,25,26,27,28]. DPP studies that have been translated into community settings have included various modifications to the original DPP protocol in order to address feasibility and sustainability challenges associated with the program, and to address the specific needs of the target communities in which studies are implemented. Examples of these modifications have included a reduced number of group sessions [11,20,22,28,29], culturally-adapted curricula [15,20,21,28,30,31], and allocation of the intervention by site rather than at the individual level for experimental study designs [12,23,32,33]. The DPP program has also demonstrated positive results in Japan, India, Finland, and China [34]. In the U.S., the DPP has been undertaken in few Asian American communities [28], none of which have been South Asian.

While diabetes prevention programs modeled after the DPP have reported beneficial results, there are few culturally-tailored programs for the Asian Indian community, and no culturally-tailored programs for South Asians exist in the U.S. [35,36]. Further, Asian Indians represent a tremendously diverse subgroup, including myriad ethnicities, religious affiliations, and linguistic groups. NYC is home to the largest Asian Indian population in the U.S. at 207,196 [37,38]. Sikhism is a religion founded in the Punjab region of India, and Sikhs are a religious and cultural subgroup with distinct practices compared to the other Asian Indian subgroups. The Sikh Asian Indian population in NYC is quickly growing, especially in the borough of Queens [39]. Although specific numbers are not known, it is estimated that the Sikh population in NYC ranges from 50,000 to 80,000 [40,41]. A significant portion of Sikh Asian Indians are limited English proficient and are uninsured. Additionally, a majority of Sikh Asian Indian men in NYC are concentrated in service sector jobs like taxi driving and construction work or are small business owners. Of further importance, Sikh Asian Indians have been the target of discrimination and harassment in the U.S. after the events of 11 September 2001, and despite their population growth and presence, there is widespread lack of understanding regarding the Sikh faith and community [40]. Studies from the U.S., Canada, and the United Kingdom have suggested that the burden of CVD and diabetes among Sikh Asian Indians is high [42,43,44]. Given the unique cultural, linguistic, and social profile of Sikh Asian Indians in NYC, culturally and linguistically tailored health promotion and prevention efforts may be important in improving health outcomes. The purpose of this study is to explore the impact, acceptability, and feasibility of a pilot community health worker (CHW) intervention designed to improve health behaviors and health outcomes related to diabetes prevention among Sikh Asian Indians identified as at-risk for diabetes who are living in NYC.

## 2. Methods

### 2.1. Framework

The research study entitled Project RICE (Reaching Immigrants through Community Empowerment), is part of the New York University (NYU) Prevention Research Center, a Centers for Disease Control and Prevention (CDC)-funded initiative rooted in the principles of community based participatory research (CBPR). In CBPR studies, diverse stakeholders with various knowledge and expertise partner to understand community concerns and develop action-oriented solutions to address them. The project was co-led by an academic partner and the largest Sikh Asian Indian American social service agency in NYC, each of which served as active and equal partners in the research process. In addition, a coalition of community leaders, researchers, health providers, and CHWs was formed, and provided input on each step of the research process to ensure the project was culturally and linguistically appropriate for the target community. The CHWs and staff at the Sikh Asian Indian community-based organization (CBO) were active members of the coalition and a unique source of community knowledge, providing critical input and guidance during all stages of the study. Table 1 summarizes each stage of the study process and how community input and leadership were integrated into the project.

### 2.2. Recruitment and Study Design

Individuals were eligible to participate in the intervention if they: (1) self-identified as Sikh Asian Indian; (2) were identified as at-risk by an interviewer-administered diabetes risk assessment tool adapted from the American Diabetes Association, which calculates “at-risk” scores based on family history of diabetes, body mass index (BMI), and other factors [45,46]; and (3) were between 18 and 75 years of age.

**Table 1:** Example of Community Engagement through the Research Process.

Stage of Research Process	Engagement of Community Partners/Representatives
Project Initiation	Academic agency conducted a health needs assessment survey in collaboration with South Asian community organizations in 2007 which highlighted high burden of diabetes in this population.
	Prior to grant submission, academic agency worked with largest Sikh social service agency in New York City to develop a diabetes prevention protocol.
	Grant submission was developed collaboratively and awarded.
	Memorandums of Understanding were developed jointly by academic and community partner outlining roles and responsibilities on the project. Grant funds are distributed across both community and academic partners.
	CBO selected key community partners (community leaders, religious leaders, health providers, media) to participate in coalition; Academic agency identified health professionals with expertise on topic areas relevant to the intervention.
Study Design	CHWs hired by the CBO were based at CBO site to build capacity within the organization and ensure acceptance by the community.
	Coalition tailored existing curricula and developed evaluation tools for use in the Sikh community, with particular importance placed upon cultural relevancy of concepts and examples, and linguistic concordance.
	Screening tool adapted to include Asian BMI categories based on input from community health providers.
	Community partner input into study design facilitated adapting the DPP for the Sikh community (e.g., reduction of the total number of sessions, translation of study materials).
	Due to concerns regarding the close-knit nature of the community within neighborhoods and negative community perceptions regarding randomization at the individual level, partners recommended that the treatment and control groups should be allocated by neighborhood.
Study Implementations	CHWs recruited participants at partner gurdwara sites as well as other community locations; collaborative meetings were held with key gurdwara leaders to ensure support and ownership for the project.
	Intervention was delivered by trained CHWs who were members of the communityData was collected by trained CHWs who were members of the community.
Analysis & Interpretation	Outcomes, challenges, and lessons learned were assessed and reviewed by the coalition in a continuous process through weekly calls and monthly meetings to adapt and refine the intervention and ensure continued cultural and linguistic relevancy.
	CBO partners and community representatives consulted to interpret data findings.
Dissemination of Findings	Held community forums and presented project findings in “lay language”.
	Dissemination of study findings to ethnic media.
	CHW Supervisor served as co-presenter at multiple public health conferences; CBO staff serve as co-authors on project publications.
Sustainability	Ongoing efforts to sustain funding for diabetes- and health promotion-related education and services so that project activities can continue beyond existing grant.
	New health priority areas identified by CBO based on formative research conducted for this project; funding sought and secured by CBO.

Based on input from health provider members of the project coalition, the diabetes risk assessment tool was modified to reflect Asian BMI categories for overweight (23–27.49) and obese (≥27.50) [47,48,49]. Participants were ineligible if they had previously been diagnosed with diabetes by a health professional, had serious health problems (e.g., terminal illness), or had participated in a previous cardiovascular disease study. CHWs recruited participants at health fairs and cultural fairs at gurdwaras (Sikh religious institutions) and other community settings between March 2012 and May 2013. The protocol was approved by the NYU School of Medicine Institutional Review Board.

**Figure 1:**
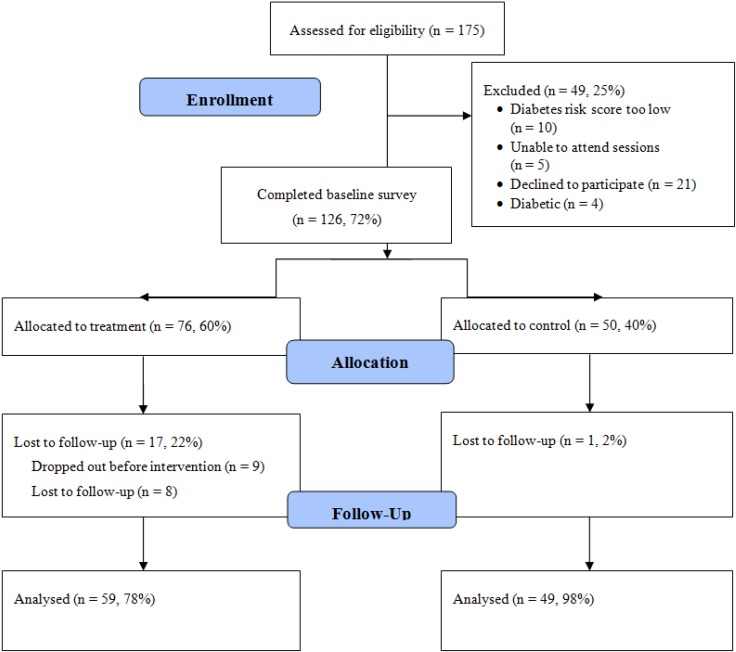
CONSORT Diagram of Study Sample.

A total of 175 individuals were screened for eligibility across the two sites and 75% were eligible and enrolled (n = 126), consenting to participate in the study and completing the baseline assessment. In order to alleviate concerns regarding contamination [50] due to the close-knit nature of the community and the concentration of Sikh Asian Indians by neighborhood, participants from one neighborhood site were allocated to the treatment group (n = 76) and participants from another neighborhood site were allocated to the control group (n = 50) (see Figure 1).

The two neighborhoods (Richmond Hills and South Ozone Park, located in the southwestern portion of the borough of Queens) were selected due to their high concentration of the target community [51]. Neighborhoods were also demographically similar. For example, Census data indicates foreign-born populations in these two neighborhoods have similar levels of education, income, and poverty level; and do not differ significantly by gender, age, Asian ethnicity, employment, or marital status [52]. Participants in the treatment arm received a multi-component CHW-led intervention, while participants in the control group were instructed to engage in standard care, including seeking preventive and acute care from their usual healthcare source as needed. Additionally, all participants assigned to the control groups were invited to receive the full intervention after serving as a control for the 6-month study period.

### 2.3. Intervention

The intervention consisted of six CHW-facilitated interactive group sessions of approximately 2 h in length and included the following topics: diabetes prevention, nutrition, physical activity, diabetes complications and other cardiovascular diseases, stress and family support, and access to health care. Sessions were held every three weeks in a convenient community setting. Treatment group participants also received follow-up phone calls from the CHWs (two calls after sessions one through five for a total of 10 calls over the 6-month intervention period), during which individualized challenges, strategies, and action plans for improving diet and physical activity and reducing stress were discussed. Following the principles of CBPR, community partner input into project design was critical in adapting the DPP for the Sikh community, resulting in the reduction of the total number of sessions from 16 to 6, and the lengthening of session time to approximately 2 h to ensure adequate time to deliver the curriculum. Sessions were held during weekend and early afternoon hours to accommodate participant schedules, particularly women with childcare obligations.

The project curriculum was adapted from existing curricula materials that have previously been validated in minority communities. These curricula include: the National Heart, Lung, and Blood Institute’s Healthy Heart Healthy Family [53], the DPP [9], the National Diabetes Education Program’s Power to Prevent (PTP) and Road to Health curricula [54], and a diabetes management curriculum used in the NYC Bangladeshi population [55]. Findings from a mixed-methods formative study were used to inform inclusion of culturally relevant topics and strategies in the curriculum. Coalition members who were health professionals, including a nutritionist, a certified diabetes educator, a physical therapist, and a mental health professional, reviewed curriculum components relevant to their areas of expertise. In addition, community partners included cultural and religious messaging to promote healthy living and overcome cultural barriers. For example, the strong tradition of community service in Sikhism was leveraged to promote preventative health. A Sikh nutritionist created recipes for healthier versions of commonly eaten Punjabi foods. All curriculum materials were developed in English, translated into Punjabi, and reviewed for accuracy by bilingual study staff. Group activities, physical exercise, culturally-appropriate images and language, and adult learning techniques were incorporated into all sessions. Session adaptation from the DPP and examples of culturally-tailored intervention components are detailed in Table 2.

**Table 2:** Adaptation and Culturally Tailored Components of Curriculum.

Project RICE Curriculum Session Title & Content	Corresponding DPP/PTP Session	Tailored Cultural Components
Diabetes prevention: Diabetes information; Prevention of diabetes; Myths and Facts about diabetes; Goal-setting.	*Welcome to the Lifestyle Balance Program* *Diabetes Overview: Part 1*	Concept of prevention tied to Sikh core values, e.g., discussion of the concept of “Saint-Soldier” in Sikhism, which promotes discipline in spiritual practice as well as in social responsibilities
		Discussion of diabetes prevalence and increased risk of diabetes in Asians
		Discussion of diabetes among Sikh Asian Indians
		Explanation of BMI and at-risk BMI in Asian communities
		Dispelling common cultural misconceptions regarding diabetes (e.g., getting diabetes is a natural part of aging)Incorporation of culturally appropriate images and language
Nutrition: Nutrition and Food; Eating a balanced diet; The Plate Method; Overcoming barriers to eating out/social situations; Reading a Nutrition Label; Goal-setting for healthy eating.	*Be a Fat Detective* *Three Ways to Eat Less Fat* *Healthy Eating* *Take Charge of What’s Around You* *Problem Solving* *Four Keys to Healthy Eating Out* *Make Social Cues Work For You* *Portion Size*	Photos of typical Punjabi/North Indian foods
		Healthy elements in traditional Indian cooking (e.g., whole grain options for rotis, incorporating fruits and vegetables)
		Identifying and limiting deep-fried snacks high in salt and sweets high in fat and sugar; substituting sweets with fruits
		Healthy vegetarian options
		Healthy versions of popular Indian recipes
		Following the Plate Method with traditional Punjabi foods
		Managing expectations for eating langar at gurdwara
		Reading food labels
		Working with women participants to improve nutrition in the entire household
		Incorporation of culturally appropriate images and language
Physical activity: Caloric intake and energy balance; Benefits and types of exercise; Injury prevention; Incorporating routines; Overcoming barriers; Practice activity and goal-setting.	*Getting Started Being Active* *Move Those Muscles* *Take Charge of What’s Around You* *Tip the Calorie Balance* *Problem Solving* *Jump Start Your Activity Plan* *Make Social Cues Work for You*	Discussion of physical activity as essential to physical and mental fitness (e.g., encouragement to practice similar discipline in physical activity as in prayer)
		Home-based exercise/activities
		Practice Activity
		Incorporation of culturally appropriate images and language
Diabetes complications and other cardiovascular diseases: Diabetes complications; Heart disease and stroke; Staying motivated and goal-setting.	*Diabetes Overview: Part 2* *Problem Solving Ways to Stay Motivated*	Discussion of diabetes complications, heart disease, stroke
		Discussion of prevention and inter-connectedness of chronic diseases
		Discussion of cholesterol and fats in diet, blood pressure and salt in diet
		Review of popular Punjabi foods high in salt and fat and limiting these foods
		Incorporation of culturally appropriate images/language
Stress and family support: Effects of stress on health; Stress and anger management; Strategies to manage depression; Family support and goal-setting.	*Talk Back to Negative Thoughts* *The Slippery Slope of Lifestyle Change* *You Can Manage Stress* *Problem Solving* *Ways to Stay Motivated* *Get Your Family and Friends Involved*	Discussion of Naam Simran, a meditation practice in Sikhism
		Progressive muscle relaxation for stress relief
		Strategies to manage depression; discussion around stigma associated with mental health (e.g., depression)
		Incorporation of culturally appropriate images and language
Access to healthcare: Communicating with the doctor; Preparing for a doctor’s visit; Accessing health services.	*Partner with Your Health Care Provider* *Ways to Stay Motivated*	Preparing for a doctor’s visit
		Communicating with the doctor
		Patient bill of rights and language access laws
		Review of NYC Health and Hospitals Corporation Options Program and the Affordable Care Act
		Health access resources and providers who speak Punjabi
		Incorporation of culturally appropriate images and language

The intervention was led by three trained, bilingual, Sikh Asian Indian CHWs and a bilingual, Asian Indian CHW supervisor at the CBO. The CHW supervisor participated in a two-part 105-h core competency and curriculum based training given between May and September 2010 [56]. The training focused on comprehensive skills training for CHWs and was facilitated by two trainers associated with an independent CHW professional association as well as academic researchers and healthcare professionals who provided trainings on community-based research and disease prevention and management. The CHW supervisor was trained on delivery of the adapted curriculum by academic partner staff. The CHW supervisor and study staff subsequently trained three additional study CHWs on the study protocol, delivery, and curriculum. Additionally, all study staff attended approximately 30 h of additional trainings on mental health, motivational interviewing/basic action planning, and other related topics.

### 2.4. Data Collection and Measures

#### 2.4.1. Quantitative Data Collection

Treatment and control group participants completed a baseline survey after consenting to be in the study, and follow-up assessments were conducted at 3-month and 6-month. Surveys were administered in Punjabi by trained interviewers. Additionally, clinical measurements were obtained at these timepoints.

Primary outcomes included weight, BMI, blood pressure, and glucose reduction, access to and utilization of care, and knowledge and practice of physical activity and healthy eating. Weight, blood pressure, and glucose and total cholesterol measurements were obtained by CHWs during baseline and 3- and 6-month follow-up visits. BMI was calculated at each timepoint using current weight and the height recorded at baseline, and Asian BMI categories were used. Waist circumference was obtained at each timepoint. Blood pressure was collected using previously validated methods for community-based settings [55,57]. Three resting blood pressure measurements approximately 2–3 min apart were collected at each timepoint using an OMRON automatic blood pressure monitor with participants in a seated position. The average of the second and third blood pressure readings was recorded as the final value. Two-hour fasting glucose and total cholesterol measurements were obtained via finger stick test.

Demographic variables and access to healthcare measures were adapted were from the Census American Community Survey (ACS) and the Behavioral Risk Factor Surveillance Survey (BRFSS) [58,59]. Self-efficacy questions related to exercise, nutrition, and health-related decisions were adapted from the Bandura Self-Efficacy Scale [60]. Questions on diabetes knowledge were adapted from the Michigan Diabetes Knowledge Scale and risk assessment questions from the American Diabetes Association (ADA) [45,61]. Questions on food behaviors such as portion control, preparation/buying, and planning, as well as intent to engage and motivators of physical activity were adapted from measurement of the behavioral objectives of a weight management intervention [62]. Additionally, treatment group participants were asked a series of questions assessing satisfaction with the intervention that were adapted from other studies. All survey questions were developed in English and translated into Punjabi by bi-lingual translators. Questions were reviewed by project coalition members for accuracy and cultural appropriateness and culturally relevant examples were integrated.

#### 2.4.2. Qualitative Data Collection

Qualitative data collection was conducted for program evaluation purposes. Qualitative interviews were conducted with the CHW supervisor on a quarterly basis by an independent evaluator during and after intervention completion to assess experiences in implementing the program, including barriers and facilitators to recruitment, retention, and diabetes prevention promotion. The lead investigator and the evaluator developed the interview questions using a review of relevant literature. In addition, a focus group with seven female treatment group participants was facilitated by the CHW supervisor to assess participant satisfaction and to obtain feedback for program improvements after the 6-month intervention period.

### 2.5. Data Analysis

#### 2.5.1. Quantitative Data Analysis

Descriptive statistics are summarized, and we compare baseline characteristics of the treatment and control groups for all individuals consented and enrolled into the intervention (n = 126). Changes in outcome variables were reported across baseline, 3-month, and 6-month for all individuals having complete data at all three timepoints. For continuous variables, mean change and standard deviation (SD) were reported; for categorical variables, total n and percent were reported. Within-group differences were assessed using paired-sample t-tests for continuous variables and chi-square tests for categorical variables; between-group significance was reported using *t*-tests of the total change across time points for continuous variables. Statistical significance was set at *p* < 0.05. Analyses were conducted using SPSS version 19.0 (IBM Corp., Armonk, NY, USA).

#### 2.5.2. Qualitative Data Analysis

Notes from CHW interviews and focus group transcripts were reviewed and coded by two of the co-authors for themes related to feasibility, acceptability, and changes in outcomes among study participants. Narrative analysis techniques were utilized whereby segments of text relating to themes were identified and core codes and secondary codes were assigned. Relationships between codes within themes were also explored. Discrepancies in coding were resolved by discussion and consensus between the two coders.

## 3. Results and Discussion

### 3.1. Quantitative Results

#### 3.1.1. Feasibility

Among treatment group participants in the final analyses, 97% (n = 57) completed four of the six intervention sessions, while 92% completed all six intervention sessions. In terms of follow-up phone calls, 85% participated in at least six of 10 follow-up phone calls with the CHW, and 69% participated in all 10 follow-up calls.

Approximately 17% of treatment group participants (n = 17) were lost to follow-up. Among these individuals, n = 9 did not attend any sessions after being enrolled, consented, and completing baseline despite numerous attempts at contact, and n = 8 were lost to follow-up due to lack of time to participate in the intervention, family obligations or work schedules, or leaving the U.S. to travel to the home country. Individuals that were lost to follow-up were not demographically different than the individuals that were retained in the treatment group. Among control group participants, only one individual was lost to follow-up.

#### 3.1.2. Efficacy

Socio-demographic characteristics and baseline clinical information is presented for the 126 individuals with baseline data enrolled into treatment and control groups (Table 3). At baseline, individuals in the treatment group were significantly more likely to be female, to have lived in the U.S. for fewer years, to have higher education levels, to have health insurance, to report their health as good or higher, and to have a lower weight.

Statistically significant improvements in clinical variables between baseline and 6-month were observed in glucose, weight, and BMI for the treatment group, but not for the control group (Table 4). Systolic and diastolic blood pressure decreased significantly for treatment and control groups, and in both groups the majority of individuals (96% in both groups) had controlled blood pressure at 6-month. Total cholesterol levels increased significantly for the treatment group between baseline and 6-month (*p* < 0.01). Additionally, significant between-group differences in the correct direction were observed for glucose, and the change in glucose between baseline and 6-month was significantly greater for the treatment group (*p* < 0.01). Between-group differences for changes in weight and BMI also approached significance, with greater changes seen in the treatment group.

**Table 3:** Baseline characteristics of participants (n = 126).

Characteristics	Treatment Group	Control Group	*p*-value
	(n = 76) n (%)	(n = 50) n (%)	
**Demographics**			
Age (years), mean (SD)	46.3 (11.6)	47.8 (9.5)	0.40
Female	73 (96.1)	29 (58.0)	<0.01
Born outside the U.S.	76 (100.0)	50 (100.0)	1.00
Years lived in U.S., mean (SD)	10.5 (6.9)	14.9 (6.8)	<0.01
Married	71 (93.4)	47 (97.9)	0.26
**Education**			<0.01
<High school	12 (16.2)	4 (8.2)	
High school/some college	43 (58.1)	42 (85.7)	
College graduate	19 (25.7)	3 (6.1)	
Speaks English not well or not at all	28 (37.8)	26 (52.0)	0.12
**Health insurance**			0.03
Uninsured	9 (13.0)	14 (31.1)	
Public/hospital card	50 (72.5)	29 (64.4)	
Private	10 (14.5)	2 (4.0)	
Fair or poor self-reported health	13 (17.6)	24 (50.0)	<0.01
**Clinical variables**			
Weight (lbs), mean (SD)	162.4 (26.6)	174.9 (23.0)	<0.01
BMI (kg/m^2^), mean (SD)	28.2 (4.0)	28.6 (3.0)	0.57
Overweight (23–27.49)	30 (39.5)	16 (32.0)	
Obese (≥27.50)	41 (53.9)	32 (64.0)	
Glucose (mg/dL), mean (SD)	112.3 (34.0)	110.7 (21.6)	0.78
Cholesterol (mg/dL), mean (SD)	152.1 (37.9)	138.9 (33.9)	0.07
Systolic BP (mmHg), mean (SD)	129.8 (15.9)	128.7 (16.7)	0.73
Diastolic BP (mmHg), mean (SD)	83.9 (9.0)	85.6 (10.8)	0.35
Hypertensive	22 (30.6)	17 (34.0)	
Pre-hypertensive	37 (51.4)	23 (46.0)	

Among the treatment group, 88.7% reported engaging in any physical activity at 6-month, compared to 3.8% at baseline (*p* < 0.01). The control group demonstrated a non-significant, smaller increase in physical activity. Both groups showed a significant increase in social interaction related to physical activity (e.g., whether participants were more likely to reach out to friends or family to engage in physical activity). However, this change was greater among the treatment group (3.6 *vs.* 1.9 at 6-month), and between-group differences in social interaction related to physical activity were also significant between baseline and 6-month (*p* < 0.01).

**Table 4:** Changes in outcomes.

Outcome Measures		Treatment (T) Group n = 54					Control (C) Group n = 48				
	n	Mean BL	Mean 3 M	Mean 6 M	*p*-value (BL–6 M)	n	Mean BL	Mean 3 M	Mean 6 M	*p*-value (BL–6 M)	T *vs.* C *p*-value
**Clinical variables**											
Weight, lbs	54	160.2 (27.7)	157.5 (26.6)	155.4 (25.4)	<0.01	48	174.8 (23.2)	169.1 (22.8)	173.7 (19.3)	0.53	0.10
BMI, kg/m^2^	54	27.8 (4.2)	27.4 (4.1)	27.0 (4.0)	<0.01	48	28.6 (3.0)	27.7 (3.0)	28.5 (2.7)	0.69	0.08
Waist circumference, inches	49	36.7 (5.9)	35.2 (4.6)	34.6 (4.2)	<0.01	42	36.7 (3.4)	35.8 (3.5)	35.4 (2.8)	<0.01	0.39
Glucose	50	114.5 (36.8)	96.7 (17.6)	88.9 (16.5)	<0.01	40	111.3 (22.0)	102.2 (19.3)	113.0 (12.0)	0.56	<0.01
Cholesterol	46	144.7 (35.7)	182.7 (43.3)	168.7 (30.5)	<0.01	40	138.5 (34.4)	111.7 (20.3)	137.3 (30.8)	0.85	<0.01
Systolic BP	51	131.6 (16.6)	118.6 (12.4)	118.2 (10.6)	<0.01	47	128.0 (16.2)	118.4 (16.1)	112.1 (12.1)	<0.01	0.47
Diastolic BP	51	83.1 (8.6)	78.4 (8.3)	78.0 (7.4)	<0.01	47	86.0 (10.5)	79.8 (8.8)	79.9 (5.6)	<0.01	0.61
Controlled BP, n (%)	51	36 (70.6)	45 (88.2)	49 (96.1)	<0.01	47	33 (70.2)	42 (89.4)	45 (95.7)	<0.01	n/a
**Physical activity**											
Any physical activity, n (%)	53	2 (3.8)	44 (83.0)	47 (88.7)	<0.01	38	15 (39.5)	27 (71.1)	19 (50.0)	0.36	n/a
Social interaction (1–4, 4 = highest)	53	1.4 (0.6)	2.4 (0.8)	3.6 (0.5)	<0.01	46	1.7 (0.7)	1.6 (0.6)	1.9 (0.6)	0.04	<0.01
**Food behaviors**											
Portion control (1–4, 4 = highest)	49	1.8 (0.8)	2.5 (0.8)	3.6 (0.7)	<0.01	46	2.9 (0.9)	2.1 (0.8)	2.7 (0.6)	0.09	<0.01
Eats brown rice often/almost always, n (%)	40	2 (5.0)	7 (17.5)	10 (25.0)	<0.01	26	0 (0.0)	0 (0.0)	0 (0.0)	1.0	n/a
**Diabetes knowledge**											
ADA diabetes knowledge Scale (0–8, 8 = highest)	51	3.6 (2.3)	5.5 (1.5)	6.5 (0.6)	<0.01	44	3.5 (2.1)	5.3 (1.5)	4.9 (1.3)	<0.01	<0.01
Michigan diabetes knowledge scale (0–7, 7 = highest)	50	1.1 (1.0)	3.1 (1.5)	3.2 (1.0)	<0.01	45	2.1 (1.2)	3.4 (1.8)	3.1 (1.0)	<0.01	<0.01

In terms of food-related behaviors, the treatment group showed a statistically significant increase in portion control from baseline to 6-month (1.8 to 3.6, *p* < 0.01), while a non-significant change was seen for the control group (2.9 to 2.7, *p* = 0.09); the between-group difference in change from baseline to 6-month was also significant (*p* < 0.01). In addition, 25% of the treatment group reported eating brown rice often or almost always at 6-month compared to 4% at baseline (*p* < 0.01); no individuals reported eating brown rice among the control, at either time point.

Diabetes knowledge increased significantly for both groups. Results were run using two scales. The treatment group showed a greater increase in knowledge for the ADA scale (6.5 *vs.* 4.8 at 6-month), while both groups showed similar improvements for the Michigan scale (3.2 *vs.* 3.1 at 6-month). The between-group differences from baseline to 6-month in both diabetes scales were statistically significant (*p* < 0.01).

#### 3.1.3. Acceptability

Overall, treatment group participants were satisfied with the intervention and found the CHWs to be useful in helping them to answer questions, facilitating access to a doctor, and changing their behaviors (Table 5). Everyone reported that their CHW treated them with respect and dignity all of the time, while lower percentages were shown for doctors (86%) and other health professionals (62%). Eighty-eight percent of participants were totally satisfied with the intervention, and everyone was at least very satisfied.

**Table 5:** Satisfaction with the CHW Program (n = 59).

Program Evaluation	Intervention Group (n = 59) n (%)
**Would you say you use the social service agency as a resource**	
A lot	54 (91.5)
Some	5 (8.5)
**How often does the following treat you with respect and dignity (great deal of the time)**	
CHW	58 (100.0)
Primary care doctor	49 (86.0)
Health professionals besides doctors	36 (62.1)
**I am able to tell the CHW things that I cannot tell my doctor**	
Strongly agree	43 (72.9)
Agree	15 (25.4)
Disagree	1 (1.7)
**The CHW helped me to change my behaviors**	
Strongly agree	54 (91.5)
Agree	5 (8.5)
**I see a doctor more often because of the CHW**	
Strongly agree	31 (52.5)
Agree	28 (47.5)
**I feel more confident asking my doctor questions because of the CHW**	
Strongly agree	32 (54.2)
Agree	27 (45.8)
**Overall, how satisfied are you with the CHW? (out of 10)**	
8–Very satisfied	1 (1.7)
9	6 (10.3)
10–Totally satisfied	51 (88.0)

### 3.2. Qualitative Results

#### 3.2.1. Feasibility

Findings from the qualitative data provided contextual insights that expand understanding of the main findings from the quantitative pilot results. Qualitative data further inform the feasibility issues that emerged during the pilot, including recruitment, retention, and the organization and implementation of the pilot. As discussed above, recruitment was facilitated by building on strong relationships with respected Sikh organizations in the community and by the principles of “seva”, which is a call for volunteerism and community service in the Sikh religion, which enabled CHWs to benefit from help provided by volunteers at recruitment events and in getting the word out about the project. The analysis of the qualitative data documented several barriers to recruitment. The main challenges to recruitment that CHW and focus group discussion revealed were: (1) Community members felt that if they were free of symptoms of diabetes, they did not need to take steps to prevent the disease; and (2) community members felt that diabetes was a natural part of aging and could not be prevented. As the CHW supervisor commented: “*People say there’s a need for the program but don’t show up. They say, “It’s not a problem for me”. Prevention is not a priority for the community; they have the attitude: “I’ll deal with it when I get the disease*”.”. The CHW supervisor further noted that community members seemed more motivated to participate in the intervention if they knew someone with diabetes or if the screening showed them to have an at-risk BMI: “*When I tell them their BMI, it is eye-opening and motivates people to join the program*”. Indeed, the reasons that focus group participants offered as their motivation to join the program was less about preventing disease than a result of pre-existing issues, such as previous health problems *(“I had some (health) problems so I thought I would go and learn something”*); the desire to lose weight *(“I had some extra weight; I came because of the weight”)*; and prior gestational diabetes or elevated blood sugar *(“I had diabetes when I was pregnant and the doctor told me I could develop diabetes; I also had borderline high blood sugar”).* Another recruitment challenge noted by the CHW supervisor was gender-related. She observed that it was difficult to enroll men into the intervention due to their long work hours.

Retention was facilitated by trust in the CHW and consistent follow-up, particularly by CHWs living within the community where the pilot intervention was held. The CHW supervisor’s comments provided insights on challenges that affected program retention. These issues included the extended travel by participants to India during the intervention period; difficulty scheduling education sessions and data collection meetings that would accommodate the participants’ schedule; childcare and family obligations, particularly among woman, that prevented several women from attending sessions on the arranged date and time; and long work hours, particularly among men, which prevented participating in multiple sessions.

In regards to intervention organization and implementation, attempts to facilitate program participation in order to address the scheduling barriers noted above included: hosting sessions in community locations convenient for participants, during weekends and mid-day when women have a break from household responsibilities and childcare, and offering session make-ups. The majority of the focus group’s comments indicated that the participants appreciated the CHWs’ consistent follow-up and willingness to offer make-up sessions: *“(The CHW) would call, remind about classes, and make sure we attended. Even when you couldn’t make it she would make it up. She’s very nice. She would make sure we attended all the sessions*”. Some of the comments offered by the participants even suggested a preference for classes more often, saying: “*It’s better to have them every 2 weeks. Whenever we come we learn something. If sessions are every 4 weeks we would forget everythin*g” and “*That’s right, the sooner you call us the quicker we will learn*”. The CHWs’ reports revealed that scheduling challenges were a frequent issue, as face-to-face meetings were required to collect the survey questionnaires and clinical measurements in addition to asking the program participants to attend six group sessions.

#### 3.2.2. Efficacy

The qualitative data also provided insight into facilitators and barriers to improving health outcomes and behaviors. A number of the comments made by the focus group participants reinforced quantitative findings that indicated the intervention improved knowledge on diabetes prevention and health behaviors, as illustrated in the following statement offered by one participant, “*I got to learn a lot and practice what I learned. I watched my diet and took care to keep diabetes away. I had borderline high sugar but I am ok now*”. Another benefit that several participants felt they received from the program was the program’s focus on prevention was helpful in increasing knowledge and motivating healthy behavior change. As one participant explained: “*A person who has high blood pressure generally knows what to eat and what not to eat. Not in too much detail though. That I found out about and experienced here. Like I used to eat a lot of sweets, I reduced that quite a bit. You get scared of developing diabetes*”. Similarly, a few participants also highlighted the value of the curriculum’s focus on monitoring health outcomes in helping to motivate them to further behavior change. This sentiment is reflected in the following observation made by one participant: “*The knowledge that we received…we were able to implement. For example, we would come to class and learn. Then we would go home and moderate our diet. Otherwise we wouldn’t pay attention and eat what’s made. Additionally there were the (blood) tests, we would find out what’s going on with us (our numbers)…*”. Qualitative data also explicated how the participants experienced the social support derived from their program participation was both an important motivator for engaging in the program and also played a role in helping some of these participants make lifestyle changes. For example, one participant shared that she initially joined “for fun” before becoming more serious about participating: *“When (the CHW) told us to attend we thought “let’s go”. We would just sit outside chatting in the evenings anyway. We just came for fun. But when we attended, we got serious”.* Similarly, another participant explained further that: *“sometimes we call each other and discuss what to eat or what not to eat. Even if we are eating it but we will tell each other not to”.*

The qualitative data also provided further illumination on the challenges that participants experienced in terms of changing health behaviors. Although many of the participants reported that the knowledge they received from the intervention was helpful, some participants also noted that their social contexts sometimes prevented them from implementing the changes learned through the intervention. As one participant observed, “*We come from big families so that's the catch*”, explaining that dietary changes were harder to make at the family level. The CHWs’ statements also contained the observed that in general goal-setting exercises were challenging for participants. As the CHW supervisor noted: “*It is hard for people to set goals. People say, “You give me a goal and I’ll do it”. But they are unwilling to set their own goals. They ask for an example, and if I give them one, they will just use the example*”.

#### 3.2.3. Acceptability

The qualitative data expanded upon the importance of cultural relevance and use of the CHW model in the acceptability of the intervention. The project CHWs shared participants’ cultural backgrounds and language, and were able to leverage their knowledge of community resources and religious networks (e.g., gurdwaras and prayer groups) to increase their outreach efforts and ensure the relevance and acceptability of the study to the community. There were a number of features of the CHW model approach, which promotes experiential- and co- learning, that participants found satisfactory. Some of the participants expressed satisfaction with the group format: “*It was good. We were sitting in a group, asking and talking with each other, we learned a lot*”. Another positive aspect of the program that a few participants commented upon was their relationship with the CHWs, some of these comments described this relationship in warm, familial terms: “*She was very, very good. She explained things in a way that made me feel like a family member*”. As is custom in the Punjabi language, all participants and CHWs referred to each other in their statements as “*bhenji*”, meaning “sister”, reflecting congruency with the principle of peer-learning inherent in the CHW model approach. Another further feature that impressed most of the participants was that the program was culturally-tailored for the Sikh community. As one participant shared: “*(The CHW) told us (the program) was made specially (sic) for our community*”. In addition, a number of participants noted that they were appreciative that this Centers for Disease Control and Prevention-funded program demonstrated an interest by the government in learning more about the Sikh community and in promoting health, as the following statements reflect: “*It was good to know (the government is) interested in getting the knowledge”* and *“The government is concerned in our health, is taking care of us*”. The project’s connection to a reputable Sikh organization in the community as well as to an academic medical school facilitated trust of the community; and securing the support of the gurdwara leadership was critical to acceptance by the community. The CHW supervisor noted: *“I’ve learned the importance of working closely with the gurdwaras, which are at the heart of the community, and where Sikhs share many meals together. We have been fortunate to have the support of the Sikh Cultural Society, Inc., a well-established gurdwara in the community”*.

## 4. Discussion

Overall, the RICE project demonstrated high feasibility, acceptability, and efficacy of a CHW-led intervention to promote diabetes prevention among at-risk individuals in the Sikh community. Our study reports high retention rates compared to other studies reported in the literature [12,33]. Participants reported positive feedback about the program and about the CHWs, particularly regarding the cultural congruence of CHW and the strong connectedness of the intervention with community resources and cultural and religious norms and values. Both quantitative and qualitative pilot findings demonstrated high appropriateness and acceptability to the target community, indicating that the pilot can be successfully translated into a full intervention.

Positive changes were seen among the treatment group between baseline and 6-month. Among treatment group participants, statistically significant changes in weight, BMI, blood pressure, glucose, physical activity, food behaviors, and diabetes knowledge were reported. Qualitative findings indicate that the culturally tailored aspect of the curriculum, consistent follow-up from the CHWs, and the integration of social support mechanisms through the program were instrumental in helping participants achieve changes in health outcomes and behaviors.

After implementation of the pilot intervention, several feasibility barriers were highlighted through our data. Congruent with CBPR methods, challenges from the pilot study have been reviewed by the community coalition and used to adapt the full intervention, which is currently being implemented. These challenges to feasibility and how they are being addressed in the full intervention are summarized in Table 6. In particular, we address the challenges associated with recruiting male participants, lack of awareness regarding prevention, challenges associated with goal-setting for participants, and challenges with retention.

**Table 6:** Challenges experienced during pilot and modifications made to the full intervention.

Challenges	Reason	Modifications Made to Full Intervention
Difficulty recruiting male participants	Sikh males are concentrated in small business and service sector position with long work hours	Working with gurdwara leadership to recruit more men into the project
		Accommodating male participant schedules through additional sessions at convenient times
		Word-of-mouth referrals—ask screening and intervention participants to refer their friends and family members
Lack of awareness regarding prevention	Misperception regarding the need for diabetes prevention in the absence of a diabetes diagnosis; general lack of community-level emphasis on prevention	Stronger partnerships and outreach to gurdwara leadership and staff to promote awareness for prevention
		Better messaging of the social and financial implications of having the disease *vs.* attending a free program
		Hold more community education events on diabetes and diabetes prevention in the Sikh American community
Goal-setting for participants	Participants were reluctant to engage in goal-setting or not familiar with the process	CHWs will receive additional training on motivational interviewing and goal-setting that is culturally tailored
Retention	Participants were unable to sustain participation due to family obligations and/or work schedules	Accommodating participants’ schedules by offering additional sessions at convenient times
		Building in more incentives/prizes for retention
		Marketing of Project RICE at routinely held health fairs to increase awareness of the program

Some limitations should also be mentioned. First, there were significant demographic differences between the treatment and control group participants, namely that the majority of treatment group participants were female, more likely to be college-educated, and had lived in the U.S. for a shorter duration than control group participants. Thus, our study findings may not be generalizable to the Sikh population. Further, the statistically significant between-group differences may be a reflection of the differences in gender, years lived in the U.S., or education levels between the groups. However, our findings also demonstrate statistically significant changes in health behaviors, outcomes, and knowledge within the treatment group, demonstrating the impact of the intervention. Our quasi-experimental design does provide a comparison arm unlike many other pilot studies that have adapted the DPP [15,17,18,20,22,26,63,64,65], and the strength of our study outcomes are encouraging. Second, though we attempted to control for contamination by allocating to group by neighborhood, control group participants did demonstrate some positive changes in terms of blood pressure control and other health behaviors, suggesting that some of the intervention health promotion efforts may have been disseminated beyond the treatment group, or that treatment group participants were motivated to make health changes. However, for most study outcomes, the treatment group demonstrated greater improvements compared to the control group. Third, our collection of clinical data on glucose and total cholesterol were limited to a two-hour fasting finger stick test, which may have limited accuracy and specificity [66]. Our study reported that participants in the treatment group experienced an increase in total cholesterol. Because the test only allowed an assessment of total cholesterol, we are not able to determine if this increase is a result in increase in HDL levels (which would be a desirable study outcome) or LDL levels. Additionally, study results for glucose demonstrated significant change in the right direction, providing important pilot data on the potential efficacy of the intervention for decreasing fasting glucose assessed through more rigorous methods. Further, our results indicate positive changes in health behaviors and weight loss, indicating the potential impact of a full intervention.

## 5. Conclusions and Implications

This study is the first to report on the results of a pilot CHW intervention to promote diabetes prevention in the Sikh Asian Indian community of NYC. As such, it fills an important gap in the literature on developing culturally-tailored interventions for underserved, high-risk, racial ethnic minority communities. Study findings indicate that the CHW model is acceptable in this community and that it helps to promote behavior changes in nutrition and physical activity, important components of diabetes prevention. Another major strength of this study is the use of both qualitative and quantitative methods to assess the feasibility, acceptability, and outcomes of the pilot intervention. Further, our study examined CHW characteristics and aspects of CHW acceptability, which have been noted as a gap in the literature [67]. Such findings contribute to the growing literature on the role of CHWs in addressing health disparities. Finally, in highlighting some of the unique challenges faced by immigrant community members with whom we partnered on the project, findings provide important insight into ways that programs can be tailored to meet the needs of minority communities.

Given that the rates of diabetes among Asian Indians are rising worldwide, as well as the tremendous diversity that is encompassed by this population, effective and culturally-tailored health care interventions are needed to mitigate the burden of diabetes in this population. The development, implementation, and evaluation of innovative programs that address local ethnic and cultural norms, build upon community assets, and are conducted with community-academic partnerships will provide important information to improve diabetes prevention programs and the health of racial ethnic minority communities.
